# Standardization in the judgment of line orientation test in elderly people in Northeast Brazil

**DOI:** 10.1590/1980-5764-DN-2024-0130

**Published:** 2024-12-02

**Authors:** Paulo Roberto de Brito-Marques, Janaina Mariana de Araujo Miranda Brito-Marques, Cácia Carolina de Carvalho Silva Miranda, Herickssen Gustavo Medeiros-Silva

**Affiliations:** 1 Universidade de Pernambuco, Faculdade de Ciências Médicas, Unidade de Neurologia Cognitiva e Comportamental, Recife PE, Brazil.; 2 Universidade de Pernambuco, Hospital Universitário Oswaldo Cruz, Recife PE, Brazil.

**Keywords:** Neuropsychological Tests, Dementia, Cerebrum, Aging, Spatial memory, Testes Neuropsicológicos, Demência, Cérebro, Envelhecimento, Memória Espacial

## Abstract

Aging is generally associated with a decline in cognition when compared to adulthood. The changes most seen are in attention, perception, working memory, short-term memory, free recall, and processing speed. Certain changes in the Judgment of Line Orientation (JLO) test may be present in elderly people.

**Objective:**

The objective of this study was to describe the difference among age, sex, schooling, Mini-Mental State (MMS), and Mini-Mental State Examination (MMSE) when compared to the JLO test in elderly people. The second is to assess the clinical use of the JLO test in elderly people's cognitive decline in Northeast Brazil.

**Methods:**

A cross-sectional, randomized study was carried out on 280 elderly people, between 60 and 89 years old, and the mean age was 69.4 (±6.8 years SD); 73.9% of the females lived in Olinda City, State of Pernambuco, Brazil. Age was stratified every 5 years between 60 and 89 years old, and schooling was divided into four subgroups between illiterate and more than 8 years of schooling. Each participant was submitted to an analysis of age, sex, schooling, risk factors, MMS, modified MMSE, and a JLO test.

**Results:**

There was no statistical difference between the sexes. However, there was a statistical difference when compared to the JLO test and age (p<0.012), schooling (p<0.001), MMS (p<0.001), and modified MMSE (p<0.001).

**Conclusion:**

We observed that with a cutoff point of 18 points, the JLO test is indicated to assess visuospatial and visuoperceptive changes in elderly people in Northeast Brazil.

## RESUMO.

O envelhecimento está geralmente associado a um declínio na cognição comparado à idade adulta. As alterações mais observadas são na atenção, percepção, memória de trabalho, memória de curto prazo, recordação livre e velocidade de processamento. Certas alterações cognitivas podem ocorrer no teste de Julgamento de Orientação de Linhas (JOL) em idosos.

**Objetivo:**

Descrever a diferença entre idade, sexo, escolaridade, Miniexame do Estado Mental (MEM) e Miniexame do Estado Mental em função da escolaridade (MEMFE), quando comparados ao teste de JOL em idosos. Avaliar o uso do teste de JOL no declínio cognitivo de idosos no Nordeste do Brasil.

**Métodos:**

Foi realizado um estudo transversal e randomizado com 280 idosos, entre 60 e 89 anos e média de idade de 69,4 (Dp±6,8 anos), 73,9% do sexo feminino residentes na cidade de Olinda, Estado de Pernambuco, Brasil. A idade foi estratificada a cada 5 anos entre 60 a 89 anos, e a escolaridade foi dividida em 4 subgrupos entre analfabetos a mais de 8 anos de escolaridade. Todos os participantes foram submetidos à análise de idade, sexo, escolaridade, fatores de risco, MEM, MEMFE e o teste de JOL.

**Resultados:**

Não houve diferença estatística entre os sexos. Porém, houve diferença estatística quando foi comparado o teste de JOL e idade (p<0,012), e escolaridade (p<0,001), e MEM (p<0,001) e MEMFE (p<0,001).

**Conclusão:**

Observamos que com ponto de corte de 18 pontos, o teste de JOL é indicado para avaliar alterações visoespaciais e visoperceptivas em idosos no Nordeste do Brasil.

## INTRODUCTION

Originally developed in 1978, the Judgment of Line Orientation Line (JLO) test remains popular in the neuropsychological assessment^1^. It is used to assess basic-level visuospatial reasoning and interpretation performance on more complex tasks that detect the intensity of types of visuoperceptive errors. It was developed by Benton "as pure a measure of one aspect of spatial thinking, as could be conceived"^1^. It is known that neuropsychological tests are considered measuring instruments and, therefore, must present some characteristics to be reliable. The characteristics considered most important relate to the validity and instrument accuracy^2^. Initially, the JLO test was developed to detect visuoperceptual and visuospatial functions typically associated with right hemisphere structures, especially parietal, occipitoparietal, and occipitotemporal structures^3^.

This test has been widely used in cognitive and neuropsychological practice for decades^4,5^, with high validity and retest reliability^6^. There have been many attempts to develop short forms^7,8^. Despite the difficulty of creating smaller shapes with JLO, the same efficiency and methods were employed to ensure its efficacy and neuropsychological safety^9–12^. This test may be also a useful tool in early screening for cognitive decline in the elderly population^1^, dementia^13^, Alzheimer's disease^14^, Parkinson's disease^15,16^, and Huntington's disease^17^. Socio-demographic and cultural factors have some relevance in the production of visuospatial tasks, especially in elderly people with lower schooling. According to sex differences, some authors’ findings suggest that males are more likely; than females, to normally attend to and be aided by geometrical reference cues^18–20^.

The population of older adults in Brazil is increasing according to *Instituto Brasileiro de Geografia e Estatística* (IBGE)^21^ (Brazilian Institute of Geography and Statistics), showing that 10.49% of the Brazilian population is 65 years old or older, with an expectation of becoming over 25% of the total population by 2058. The rate of dementia in the elderly population in Brazil is 8%^22^. Life expectancy also increased in Brazil as children, who were born in 2022, are expected to live 77.19 years^21^. The lower average scores relate to cultural diversity, especially in the outskirts of large cities^23^. Aging is generally associated with a decline in cognition compared to adulthood. The most seen changes are in attention, perception, working memory, short-term memory, free recall, processing speed, and generating and transforming spatial (nonverbal) information^12,24^.

Low schooling level is associated with significantly lower performance on visual sustained attention, learning and episodic memory, reaction time, and spatial working memory^25^. Education interacts with the gender to promote a stable performance on "commission errors" which occurs earlier in men than in women^25,26^. The objective of this study is to assess the clinical use of the JLO test in elderly people's cognitive decline in Northeast Brazil.

## METHODS

### Study design

A cross-sectional, randomized study was carried out in an elderly population living in a community in the city of Olinda, in the State of Pernambuco, Brazil. The sample, containing 253 individuals, was based on the following data:

- The neighborhood's streets were listed and drawn, using an updated map.
- On each street, all participants aged between 60 and 92 were tested, complying with the research inclusion criteria.

The sample size was calculated based on a pilot observation, a population SD of 3.8, a confidence interval of 95%, and a precision of 0.5, obtaining a total of 246 participants. Adding a margin of 3%, to prevent a possible dropout, the sample size resulted in 253 participants. The population consisted of 280 elderly people aged between 60 and 89 years, and the average age was 69.4 (±SD 6.8 years); 73.3% were females. Of the total number of the sample, 98.5% were right-handed. Participants were stratified by age into six subgroups: 60–64, 65–69, 70–74, 75–79, 80–84, and 85–89. Schooling was divided into four subgroups: illiterate, between 1 and 4 years, between 5 and 8 years, and more than 8 years. All participants underwent an evaluation on age, sex, education, MMS^27^, and the modified MMSE^28,29^ and compared to the JLO^1^. The research was authorized by the local ethics committee. The confidentiality of the study was guaranteed by those responsible for the omission of the participants’ records. Exclusion criteria were uncorrected visual and auditory deficits, neurological and psychiatric disorders, or joint disease that hindered motricity, and participants with intellectual disabilities were differentiated from illiterates by not recognizing colors, use of money, and how to use a can opener.

### Procedure

**Figure 1:**
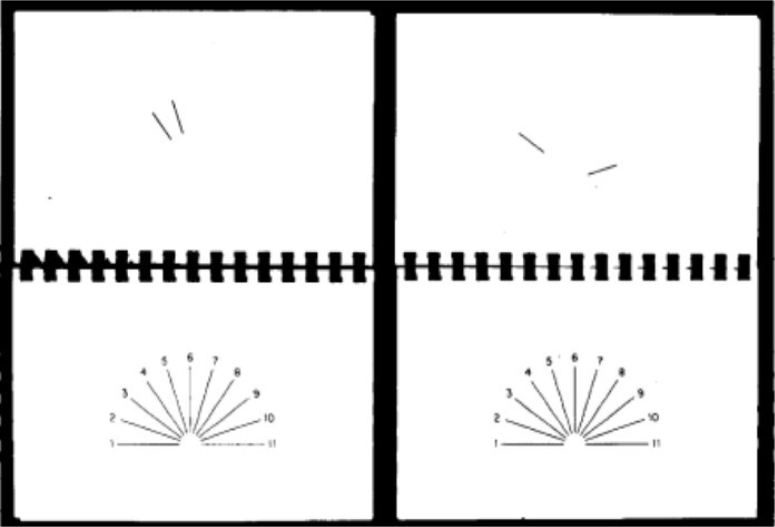
For two items of "partial lines" test, the patient identifies the slope of lines of display of 11 lines^1^.

The task used was the short JLO test (Figure 1)^1,7,8,10^. It was applied at home without interruptions. Two practice items and 15 test items were presented to each participant and assessed by an examiner. Each item consisted of a stimulus represented by a pair of lines located at the top of the card and a choice response with 11 lines, distributed in a semicircle, which appeared at the bottom of the card. Each subject identified the orientation of two lines (length=1.9 cm) by finding the lines that had the same orientation in 11 matrices of lines (length=3.8 cm) numbered 1–11, each separated by an angle of 180. The beginning of the test was preceded by five items, containing the same pairs of lines, but with larger sizes than conventional ones, and the same matrices of 11 lines. Utterance: Take a good look at these two lines (point to the target lines). Demonstrate where among the 11 lines (model lines) are oriented the same way as these two target lines and form the same angle.

### Scoring systems

The participants performed the shortened form of the JOL test evaluated by neurologists, psychologists, and speech therapists trained in cognition, and it was scored by the study coordinator. The performance of the participants was submitted to a global score analysis, where the participant was asked to match the two stimulus lines with the corresponding ones on the choice-answer card, speaking and pointing the numbers of lines, and each correct answer counted as 1 point, then the total of up to 15 points was multiplied by 2 to reach a maximum of 30 points, in addition to adding the corrected value for age and sex to the result. Points added for men: zero for younger than 65 years old, 1 point for between 65 and 74 years, and 3 points for over 75 years old. Points for women: 2 points for those under 65 years old, 3 points for those between 65 and 74 years old, and 4 points for over 75 years old. The proposed norms allow the classification of participants as defective (mildly, moderately, or severely), borderline, low normal, normal, high normal, or superior. According to Benton et al. (1994), the cutoff point is 20 points, a score between 17 and 20 represents a mild to moderate deficit, respectively, and a score below 17 points is indicative of severe impairment^14^.

### Statistical analyses

Percentiles were calculated for the JLO points, and the median was used as the cutoff point to divide the JLO variable into two groups. It was also of interest to calculate the percentiles of the JLO points considering the different age groups, as well as the schooling levels. To investigate the presence of a statistically significant association between JLO, previously categorized in up to 18 points and more than 18 points, and the variables on age, sex, schooling, MMS, and modified MMSE, the qualitative/categorical variables were used. Independently of the use of the chi-square test or Fisher's exact test (used when necessary), and in the case of quantitative/numerical variables, the Mann-Whitney test was used.

## RESULTS

### Performance of subjects

Demographic data are shown in Table 1 as age, schooling, MMS, and modified MMSE, including the cutoff point. In the personal history, cardiovascular risk factors were found in the population, we highlight that 136 elderly people were hypertensive, 41 were diabetic, elderly people had heart disease, and 27 reported having depression although there was no statistical difference among them. Besides education, MMS, and modified MMSE had significant associations with JLO.

**Table 1:** General distribution of demographic variables and percentiles, besides education, Mini-Mental State Examination, and modified Mini-Mental State Examination had significant associations with the judgment of line orientation test.

		Judgment of line orientation test				Total		p-value*
		Up to 18 points (n=148)		More than 18 points (n=132)				
		n	%	n	%	n	%	
Sex								
	Male	32	21.6	41	31.1	73	26.1	
	Female	116	78.4	91	68.9	207	73.9	0.073
Age in years (mean ± SD)		70.4±7.3		68.3±6.1		69.4±6.8		0.012*
	60–64	39	26.4	37	28.0	76	27.1	
	65–69	37	25.0	54	40.9	91	32.5	
	70–74	32	21.6	22	16.7	54	19.3	
	75–79	21	14.2	11	8.3	32	11.4	
	80–84	11	7.4	5	3.8	16	5.7	
	85 and more	8	5.4	3	2.3	11	3.9	0.037
Education in years (mean ± SD)		3.5±2.6		5.2±3.5		4.3±3.2		<0.001†
	Illiterate	21	14.2	9	6.8	30	10.7	
	1–4	77	52.0	54	40.9	131	46.8	
	5–8	44	29.7	47	35.6	91	32.5	
	More than 8	6	4.1	22	16.7	28	10.0	0.001
MMSE (mean ± SD)		21.1 ± 4.6		24.1 ± 2.9		22.5 ± 4.1		<0.001*
Modified MMSE (mean ± SD)		23.5 ± 5.0		26.4 ± 2.3		24.9 ± 4.2		<0.001*

Abbreviations: MMSE: Mini-Mental State Examination.

Notes:

* χ^2^ test (or Fisher's exact test when necessary);

† Mann-Whitney test.

## DISCUSSION

In the current study, the cutoff point for the JLO test was 18 points close to that found in Benton et al. study^1^. This test proved to be a neuropsychological instrument of great breadth, overcoming cultural barriers over time^1,6-8,10^. However, the culture was not sufficient to neutralize the results obtained in the JLO test while comparing to the studies^1,6–8^. According to the cutoff point between our results and Benton's test, the JLO test could be used in the Northeast of Brazil as a neuropsychological instrument in elderly people with cognitive decline or visuospatial or/and visuoperceptive-specific changes. The use of the JLO test can show early changes, especially in neuropsychological practice and cognitive neurology in elderly people. In a Spanish study, the JLO test was also compared with other neuropsychological tests differentiating active and non-active elderly people^30^.

Males and females did not present significant differences in the JLO test. However, when thinking about sex differences in cognitive abilities, the question that is usually asked is whether males and females are similar or different. The answer to questions about cognitive sex differences is not whether the sexes are similar or different because both females and males are similar and different in many ways^18–20^.

Controlling risk factors is still a complication in developing countries. Blood pressure is the most common risk factor in the elderly population in Brazil^21^. Although blood pressure associated with increased cholesterol is a risk for Alzheimer's disease in middle-aged people, with clinical repercussions at senile age^31^, there was no association between any risk factor, including arterial hypertension in the JLO test.

The effect of age on attentional skills and psychomotor performance remains, however, poorly understood. All of its cognitive domains may not have the same degree of equality in aging, and the efficiency of attentional processes often depends on task conditions and emotional stability^24^. In our study, the elderly people aged between 65 and 70 presented better results than those between 60 and 64 years old. Although the neuropsychological assessment took place in their homes, giving them more peace of mind, the elderly people are a vulnerable age group and feel threatened when something new occurs to them. Other factors besides aging may have some relevance in worsening among younger elderly participants between 60 and 64 years old, such as lower general knowledge and life experience, quality of education, and cultural factors^32^.

The quality of education in public schools in Brazil continues to be a limiting factor in the development of the middle and low-income population^1,23,33,34^. In our study, the schooling presented a direct regression on the JLO test percentiles. The lowest percentiles were 13 and 18 for the illiterate and 1–4 years schooling group, respectively, and the highest was 24 for those with more than 8 years of schooling. It showed that the schooling level had a direct influence on the results in visuospatial perception in the JLO test.

The MMS is the most used test for cognitive screening in the world, including Brazil^28,29,31,34,35^. It was observed that to reach the cutoff point of the JLO test, elderly people needed to exceed the average of 22 points for the MMS and 25 points for the modified MMSE, respectively^29,31^. It is possible that these lower-than-expected MMS results could indicate a pathway to dementia if prevention had not been undertaken^22,36–38^, including Alzheimer's disease^39^.

The main limitation of the study was that this was a cross-sectional study. The difficulties that may have arisen from senility could have changed the course of the study if the participants were followed. Therefore, a longitudinal study could identify cases with dementia and early Alzheimer's disease. We should be recognized as a study limitation since the median may not reflect the maximum sensitivity or specificity for clinical tests.

In conclusion, we observed that with a cutoff point of 18 points on the JLO test, it proved to be a neuropsychological instrument to be used among elderly people and with different schooling levels associated with or without MMSE and modified MMSE. For elderly people over 75 years old with schooling up to 4 years, the JLO test may indicate alterations compatible with cognitive decline and perhaps a small percentage of people with silent neurodegenerative dementias. We also conclude that these JLO short-form normative data may be used in clinical neurology screening situations or when serial assessments are needed.
